# Supplementary material for: Efficacy of Different Testing Scenarios in Reducing Transfusion-Transmitted Hepatitis B Virus (TT-HBV) Infection Risk
Source: Viruses. 2022 Oct 15;14(10):2263. doi: 10.3390/v14102263 (PMC9609221; doi:10.3390/v14102263)
Supplement: Supplementary file 1 [file viruses-14-02263-s001.zip › viruses-1954196-supplementary.pdf]

Supplementary Tables - manuscript viruses-1954196
Lelie et al - Efficacy of HBV testing scenarios

Supplementary table S1. Comparison of Ultrio Plus to Ultrio HBV NAT yield rates observed in sequential screening periods and calculated enhancement factors per infection stage and donation category in South-Africa [7] and Hong Kong [8] (between brackets corrected for HBsAg prevalence reduction factor).

**Blood center (country)**	**HKRCBS (Hong Kong)**			**SANBS (South Africa)**		
**Prevalent HBV genotypes**	**genotype B, C**			**genotype A1**		
	**yield (rate/million)**		**UP/U yield rate factor (..)^**	**yield (rate/million)**		**UP/U yield rate factor (..)^**
**NAT assay screening period**	**Ultrio (U)**	**Ultrio Plus (UP)**		**Ultrio (U)**	**Ultrio Plus (UP)**	
**Frist time donations**	**92551**	**80565**		**90871**	**98656**	
Window period	4 (43.2)	4 (49.6)	1.15 (1.86)	9 (99.0)	19 (192.6)	1.94 (2.04)
HBsAg+/DNA+	1550 (16747.5)	866 (10749.1)	0.64 (1.03)	697 (7670.1)	742 (7521.1)	0.98 (1.02)
HBsAg+/DNA-	143 (1545.1)	43 (533.7)	0.35 (0.56)	34 (374.2)	16 (162.2)	0.43 (0.45)
all HBsAg	1693 (18292.6)	909 (11282.8)	0.62	731 (8044.4)	758 (7683.3)	0.96
OBI	12 (129.7)	27 (335.1)	2.58 (4.19)	44 (484.2)	79 (800.8)	1.65 (1.73)
OBI to WP NAT yield rate factor	3.0	6.8		4.9	4.2	
**Lapsed + Repeat donations**	**424521**	**318761**		**684530**	**691235**	
Window period	9 (21.2)	15 (47.1)	2.22 (2.82)	36 (52.6)	53 (76.7)	1.46 (1.58)
HBsAg+/DNA+	99 (233.2)	64 (200.8)	0.86 (1.09)	85 (124.2)	83 (120.1)	0.97 (1.05)
HBsAg+/DNA-	16 (37.7)	4 (12.5)	0.33 (0.42)	4 (5.84)	0 (0.0)	0.0 (0.0)
all HBsAg	115 (270.9)	68 (213.3)	0.79	89 (130.0)	83 (120.1)	0.92
OBI	89 (209.6)	136 (426.6)	2.04 (2.58)	50 (73.0)	83 (120.1)	1.64 (1.78)
OBI to WP NAT yield rate factor	9.9	9.1		1.3	1.4	
**All donations**	**517072**	**399326**		**775401**	**789891**	
Window period	13 (25.1)	19 (47.6)	1.89 (2.70)	45 (58.0)	72 (91.15)	1.57 (1.56)
HBsAg+/DNA+	1649 (3189.1)	930 (2328.9)	0.73 (1.04)	782 (1008.5)	825 (1044.5)	1.04 (1.03)
HBsAg+/DNA-	159 (307.5)	47 (117.7)	0.38 (0.55)	38 (49.0)	16 (20.3)	0.41 (0.41)
all HBsAg	1808 (3496.6)	977 (2446.6)	0.70	820 (1057.5)	841 (1064.6)	1.01
OBI	101 (195.3)	163 (408.2)	2.09 (2.99)	94 (121.2)	162 (205.1)	1.69 (1.68)

^ between bracket is Ultrio Plus to Ultrio yield rate enhancement factor corrected for HBsAg prevalence reduction factor

Supplementary table S2. Projected yield rates of five infection stages (HBV marker profiles) per million donations that are expected to be detected by Ultrio Plus ID-NAT and HBsAg PRISM in four geographical regions based on observed yield rates with Ultrio ID-NAT in these regions [3] and reported Ultrio Plus over Ultrio NAT yield enhancement factors in South Africa [7] and Hong Kong [8] (see Table S1).

		Frist time donations		Lapsed + Repeat donations		All donations	
**Infection stage**	Code yield rate (U)#	UP/U factor	Projected yield rate (UP) per million	UP/U factor	Projected yield rate (UP) per million	UP/U factor	Projecte d yield rate (UP) per million
**South Africa**		n=360818		n=3210497		n=3571315	
Pre-HBsAg WP NAT yield	b_u	2.04b_u	147.0	1.58b_u	43.8	1.56b_u	50.2
HBsAg+/DNA+	c_u	c_u +0.59f_u	7459.7	c_u +0.59f_u	101.3	c_u +0.59f_u	844.7
Post-HBsAg WP NAT yield	d_u	1.67d_u	97.2	1.67d_u	8.8	1.67d_u	17.8
HBsAg+/DNA-	f_u	0.41f_u	114.8	0.41f_u	0.3	0.41f_u	11.8
OBI NAT yield	g_u	1.73g_u	445.9	1.78g_u	56.0	1.68g_u	91.3
total infections			8264.6		210.2		1015.8
**SE Asia**		n=324665		n=726716		n=1051381	
Pre-HBsAg WP NAT yield	b_u	1.86b_u	63.0	2.82b_u	54.3	2.70b_u	64.2
HBsAg+/DNA+	c_u	c_u +0.45f_u	9891.1	c_u +0.45f_u	191.6	c_u +0.45f_u	3186.8
Post-HBsAg WP NAT yield	d_u	4.20d_u	38.8	4.20d_u	23.1	4.20d_u	28.0
HBsAg+/DNA-	f_u	0.55f_u	565.8	0.55f_u	18.9	0.55f_u	187.8
OBI NAT yield	g_u	4.19g_u	554.9	2.58g_u	418.9	2.99g_u	463.6
total infections			10757.8		531.8		3692.8
**Mediterranean&**		n=290042		n=1806690		n=2118287	
Pre-HBsAg WP NAT yield	b_u	2.04 b_u	42.2	1.58b_u	12.2	1.56b_u	14.7
HBsAg+/DNA+	c_u	c_u +0.59f_u	1877.2	c_u +0.59f_u	15.0	c_u +0.59f_u	269.9
Post-HBsAg WP NAT yield	d_u	1.67d_u	23.0	1.67d_u	5.5	1.67d_u	7.1
HBsAg+/DNA-	f_u	0.41f_u	46.6	0.41f_u	0.5	0.41f_u	6.8
OBI NAT yield	g_u	1.73g_u	143.2	1.78g_u	108.4	1.68g_u	107.9
total infections			2132.2		141.7		406.3
**ECN Europe&**		n=294367		n=2323315		n=2617682	
Pre-HBsAg WP NAT yield	b_u	2.04 b_u	6.9	1.58b_u	3.4	1.56b_u	3.6
HBsAg+/DNA+	c_u	c_u +0.59f_u	1483.3	c_u +0.59f_u	5.0	c_u +0.59f_u	171.2
Post-HBsAg WP NAT yield	d_u	1.67d_u	0.0	1.67d_u	0.0	1.67d_u	0.0
HBsAg+/DNA-	f_u	0.41f_u	25.1	0.41f_u	0.2	0.41f_u	3.0
OBI NAT yield	g_u	1.73g_u	29.4	1.78g_u	27.6	1.68g_u	26.3
total infections			1544.6		36.1		204.1

U= Ultrio UP=Ultrio Plus

if x = parameter for Ultrio Plus then x_u is parameter for Ultrio

Supplementary Table S3. HBV transmission risk for different blood components and NAT screening systems calculated using Weusten risk models [4,10] and expressed as a percentage of the HBV-NAT yield rate (detected by Ultrio Plus or ID-NAT assay of equivalent sensitivity) for WP and OBI donations respectively [9].

Screening system	WP transmission risk expressed as a percentage of WP ID-NAT (Ultrio Plus) yield rate MID_{50} 3.16 (range 1 -10 HBV-DNA copies per component)			
	RBC 10 mL plasma	RBC 20 mL plasma	PC 50 mL plasma	FFP 200 mL plasma
cobas MPX ID-NAT^	30.9 (16.6-48.4)%	41.2 (24.7-59.5)%	55.7 (37.6-74.3)%	78.1 (59.5-96.7)%
Ultrio Plus ID-NAT#	45.9 (30.0-63.6)%	56.5 (39.3-74.7)%	71.0 (52.8-89.5)%	93.3 (74.7-111.9)%
cobas MPX MP6-NAT	59.9 (42.3-78.2)%	70.9 (52.8-89.3)%	85.6 (67.1-104)%	108 (89.3-126)%
Ultrio Plus MP16-NAT	91.5 (74.4-109.6)%	102.3 (84.6-120.7)%	117 (98.6-126)%	139 (121-158)%
HBsAg PRISM$	146 (130-164)%	156 (139-175)%	171 (153-189)%	193 (175-212)%
Screening system	OBI transmission risk expressed as a percentage OBI ID-NAT (Ultrio Plus) yield rate* MID_{50} 316 (range 100 -1000 HBV-DNA copies per component)			
	RBC 10 mL plasma	RBC 20 mL plasma	PC 50 mL plasma	FFP 200 mL plasma
cobas MPX ID-NAT^	0.76 (0.25-2.2)%	1.5 (0.5-4.0)%	3.3 (1.2-7.8)%	9.0 (4.0-14.6)%
Ultrio Plus ID-NAT#	2.9 (1.0-7.5)%	5.2 (1.9-12.3)%	10.5 (4.3-20.4)%	22.6 (12.3-31.4)%
cobas MPX MP6-NAT	4.7 (1.6-12.3)%	8.5 (3.1-19.9)%	17.1 (7.0-32.3)%	35.5 (19.9-47.4)%
Ultrio Plus MP16-NAT	12.5 (5.0-26.2)%	19.9 (8.8-37.2)%	33.3 (17.1-52.5)%	56.1 (37.2-68.7)%
HBsAg PRISM$	21.2 (10.9-36.9)%	29.9 (16.4-48.6)%	44.5 (26.6-64.5)%	68.0 (48.6-80.5)%

^ 50% and 95% LOD cobas MPX 1.6 and 7.5 copies/mL according to Galel et al [13]

50% and 95% LOD of Ultrio Plus 4.1 and 41.2 copies/mL according to Vermeulen et al [12]

$ HBsAg PRISM seroconversion point at S/CO = 960 copies/mL [6]

* assuming 50% of OBI NAT yield being anti-HBs positive (>10 mIU/mL) and not infectious [9,10]

Supplementary Table S4 with Erratum . Recalculation of probability of HBV transmission by RBC and FFP transfusion of HBsAg positive donations that are missed by HBV-DNA screening using two datasets

Item/Parameter	dataset Egypt	dataset South-Africa
Dataset from manuscript:	JVH 2022;29:330	Transfusion 2013;53:2459
50% LOD in copies/mL on Eurohep genotype A standard dilutions tested in 96 and 12 replicates in the two papers respectively	3.6	4.5
95% LOD in copies/mL on Eurohep genotype A standard dilutions tested in 96 and 12 replicates in the two papers respectively	40.1	43.1
Number of HBsAg positive donors in study	187	?
Number of HBsAg positive donations that were initially HBV-DNA nonreactive with previous less sensitive Ultrio assay	23	32
Number of Ultrio Plus replicate tests performed for viral load estimation on HBsAg positive donor samples that were initially nonreactive in previous less sensitive Ultrio assay	25	12
Number of HBsAg positive donor samples with less than 100% Ultrio Plus reactivity on replicate testing (number of potentially ID-NAT nonreactive HBsAg carrier donors)	17	25
Number of HBsAg positive donor samples with equal or less than 50% Ultrio Plus reactivity on replicate testing (number of ID-NAT nonreactive HBsAg carrier donors that likely will be observed if Ultrio Plus instead of the previous Ultrio version had been used for donor screening)	10	10
Imputed viral load in copies/mL in one HBsAg+ sample with 0/25 or 0/12 Ultrio Plus reactivity respectively	0.1	0.1
Lowest estimated observed viral load in copies/mL with 1/25 or 1/12 Ultrio Plus reactive results respectively	0.3	0.6
Highest estimated observed viral load in copies/mL with 24/25 or 11/12 Ultrio Plus reactive results respectively	47.2	24.2
Log viral load at 25% percentile (Q1) for Gumbel viral load distribution analysis in Weusten risk model (Transfusion 2017;57:841-849).	-0.003	0.180
Log viral load at 75% percentile (Q3) for Gumbel viral load distribution analysis in alternative Weusten risk model (Transfusion 2017;57:851-849)	1.115	0.982
50% infectious dose (ID_{50}) of HBV virions (or HBV-DNA copies) estimated in late acute declining HBsAg positive phase in animal infectivity experiments reviewed in JVH paper of El Ekiaby et al.	316	316
Estimated volume (mL) of plasma in RBCs	20	20
Estimated volume (mL) plasma in FFP	200	200
Sum of probabilities of HBV transmission by RBC transfusion in HBsAg positive donations with less than 100% Ultrio Plus reactivity according to Weusten formula (Transfusion 2011;51:203-15)	0.93	1.80
Sum of probabities of HBV transmission by FFP transfusion in HBsAg postive donations with less than 100% Ultrio Plus reactivity according to Weusten formula (Transfusion 2011;51:203-15)	4.57	7.14
Average probability of HBV transmission by RBC transfusion in HBsAg positive units with less than 100% Ultrio Plus reactivity (0.93/17 and 1.80/25 respectively)	5.5%	7.2%
Average probability of HBV transmission by FFP transfusion in HBsAg positive units with less than 100% Ultrio Plus reactivity (4.57/17 and 7.14/25 respectively)	27%	29%
Probability of HBV transmission by RBC transfusion based on Gumbel distribution parameters of viral loads in alternative Weusten risk model (Transfusion 2017;57:851-849)	4.7%	6.4%
Probability of HBV transmission by FFP transfusion based on Gumbel distribution parameters of viral loads in alternative Weusten risk model (Transfusion 2017;57:851-849)	23%	31%
Probability of an HBsAg positive unit to transmit HBV by RBC transfusion (0.93/187)	0.5%	?
Probability of an HBsAg positive unit to transmit HBV by FFP transfusion (4.57/187)	2.4%	?

Erratum belonging to Supplementary Table S4

In the recently published paper of Ekiaby et al [11] it was reported that the HBV transmission risk posed by HBsAg positive blood components from Egyptian donors missed ID-NAT screening was estimated at 9-45% for 20-200 mL of transfused plasma assuming a 50% infectious dose (ID_{50}) of 316 HBV virions. However when recalculating this risk for the Ultrio Plus assay it should have been 5.5 - 27% for 20-200 mL plasma. These proportions were reported to be 15% and 62% for HBsAg positive but ID-NAT nonreactive RBC and FFP transfusions respectively by Weusten et al [10] when using an alternative risk model based on viral load distribution. However the average probability in 25 South-African HBsAg positive units that could potentially be missed by ID-NAT Ultrio Plus screening was 7.2% and 29% for RBC and FFP transfusions respectively. Recalculation with the Weusten viral load distribution risk model shows that these percentages were estimated at 6.4% and 31% respectively.

Supplementary Table S5. TT-HBV transmission risk per million donations in eight HBV infection stages estimated with Weusten WP and OBI risk models for different regions and screening scenarios

South-Africa

**Infection risk category**	**First time**	**LPD+ RPT**	**All**	**First time**	**LPD+ RPT**	**All**
	**cobas MPX ID-NAT**			**Ultrio Plus ID-NAT**		
Pre-NAT WP residual risk	60.6	18.0	20.7	83.1	24.7	28.4
Pre-HBsAg WP NAT yield	169.5	50.5	57.9	147.0	43.8	50.2
HBsAg+/DNA+	7464.2	101.3	845.2	7459.7	101.3	844.7
Post-HBsAg WP NAT yield	32.8	3.0	6.0	29.2	2.7	5.3
Post NAT WP residual risk	1.5	0.1	0.3	5.1	0.5	0.9
HBsAg+/DNA-	2.4	0.0	0.2	6.9	0.0	0.7
OBI NAT yield	150.3	18.9	30.8	133.8	16.8	27.4
OBI NAT residual risk	6.7	0.8	1.4	23.2	2.9	4.7
total infectivity risk	7887.8	192.7	962.4	7887.8	192.7	962.4
	**cobas MPX MP6-NAT**			**Ultrio Plus MP16-NAT**		
Pre-NAT WP residual risk	104.2	31.1	35.6	150.4	44.8	51.4
Pre-HBsAg WP NAT yield	125.8	37.5	43.0	79.7	23.7	27.2
HBsAg+/DNA+	7455.6	101.3	844.3	7444.8	101.3	843.2
Post-HBsAg WP NAT yield	26.0	2.4	4.7	14.9	1.4	2.7
Post NAT WP residual risk	8.3	0.8	1.5	19.3	1.8	3.5
HBsAg+/DNA-	11.0	0.0	1.1	21.8	0.0	2.2
OBI NAT yield	119.1	15.0	24.4	68.2	8.6	14.0
OBI NAT residual risk	37.9	4.8	7.8	88.7	11.1	18.2
total infectivity risk	7887.8	192.7	962.4	7887.8	192.7	962.4

South East Asia

Infection risk category	First time	LPD+ RPT	All	First time	LPD+ RPT	All
	**cobas MPX ID-NAT**			**Ultrio Plus ID-NAT**		
Pre-NAT WP residual risk	26.0	22.4	26.5	35.6	30.7	36.3
Pre-HBsAg WP NAT yield	72.7	62.6	74.0	63.0	54.3	64.2
HBsAg+/DNA+	9913.2	192.4	3194.1	9891.1	191.6	3186.8
Post-HBsAg WP NAT yield	13.1	7.8	9.4	11.6	6.9	8.4
Post NAT WP residual risk	0.6	0.3	0.4	2.0	1.2	1.5
HBsAg+/DNA-	11.9	0.4	3.9	33.9	1.1	11.3
OBI NAT yield	187.0	141.2	156.2	166.5	125.7	139.1
OBI NAT residual risk	8.3	6.3	7.0	28.9	21.8	24.1
total infectivity risk	10232.7	433.4	3471.6	10232.7	433.4	3471.6
	**cobas MPX MP6-NAT**			**Ultrio Plus MP16-NAT**		
Pre-NAT WP residual risk	44.7	38.5	45.5	64.5	55.6	65.7
Pre-HBsAg WP NAT yield	53.9	46.5	55.0	34.2	29.4	34.8
HBsAg+/DNA+	9870.7	190.9	3180.0	9817.6	189.2	3162.4
Post-HBsAg WP NAT yield	10.4	6.2	7.5	5.9	3.5	4.3
Post NAT WP residual risk	3.3	2.0	2.4	7.7	4.6	5.6
HBsAg+/DNA-	54.3	1.8	18.0	107.5	3.6	35.7
OBI NAT yield	148.2	111.9	123.8	84.9	64.1	70.9
OBI NAT residual risk	47.2	35.6	39.4	110.4	83.4	92.2
total infectivity risk	10232.7	433.4	3471.6	10232.7	433.4	3471.6

LPD + RPT = lapsed plus repeat

Supplementary Table S5 continued.

Mediterranean

Infection risk category	First time	LPD+ RPT	All	First time	LPD+ RPT	All
	**cobas MPX ID-NAT**			**Ultrio Plus ID-NAT**		
Pre-NAT WP residual risk	17.4	5.0	6.1	23.8	6.9	8.3
Pre-HBsAg WP NAT yield	48.7	14.1	17.0	42.2	12.2	14.7
HBsAg+/DNA+	1879.0	15.1	270.1	1877.2	15.0	269.9
Post-HBsAg WP NAT yield	7.8	1.9	2.4	6.9	1.7	2.1
Post NAT WP residual risk	0.3	0.1	0.1	1.2	0.3	0.4
HBsAg+/DNA-	1.0	0.0	0.1	2.8	0.0	0.4
OBI NAT yield	48.2	36.5	36.3	42.9	32.5	32.4
OBI NAT residual risk	2.1	1.6	1.6	7.4	5.6	5.6
total infectivity risk	2004.6	74.3	333.8	2004.6	74.3	333.8
	**cobas MPX MP6-NAT**			**Ultrio Plus MP16-NAT**		
Pre-NAT WP residual risk	29.9	8.7	10.4	43.2	12.5	15.1
Pre-HBsAg WP NAT yield	36.1	10.5	12.6	22.9	6.6	8.0
HBsAg+/DNA+	1875.5	15.0	269.6	1871.1	15.0	269.0
Post-HBsAg WP NAT yield	6.1	1.5	1.9	3.5	0.8	1.1
Post NAT WP residual risk	2.0	0.5	0.6	4.6	1.1	1.4
HBsAg+/DNA-	4.5	0.0	0.7	8.9	0.1	1.3
OBI NAT yield	38.2	28.9	28.8	21.9	16.6	16.5
OBI NAT residual risk	12.2	9.2	9.2	28.5	21.6	21.5
total infectivity risk	2004.6	74.3	333.8	2004.6	74.3	333.8

Central North-Europe

Infection risk category	First time	LPD+ RPT	All	First time	LPD+ RPT	All
	**cobas MPX ID-NAT**			**Ultrio Plus ID-NAT**		
Pre-NAT WP residual risk	2.9	1.4	1.5	3.9	1.9	2.0
Pre-HBsAg WP NAT yield	8.0	3.9	4.1	6.9	3.4	3.6
HBsAg+/DNA+	1484.2	5.0	171.3	1483.3	5.0	171.2
Post-HBsAg WP NAT yield	0.0	0.0	0.0	0.0	0.0	0.0
Post NAT WP residual risk	0.0	0.0	0.0	0.0	0.0	0.0
HBsAg+/DNA-	0.5	0.0	0.1	1.5	0.0	0.2
OBI NAT yield	9.9	9.3	8.9	8.8	8.3	7.9
OBI NAT residual risk	0.4	0.4	0.4	1.5	1.4	1.4
total infectivity risk	1505.9	20.0	186.3	1505.9	20.0	186.3
	**cobas MPX MP6-NAT**			**Ultrio Plus MP16-NAT**		
Pre-NAT WP residual risk	4.9	2.4	2.5	7.1	3.5	3.7
Pre-HBsAg WP NAT yield	5.9	2.9	3.1	3.8	1.8	1.9
HBsAg+/DNA+	1482.3	5.0	171.1	1480.0	5.0	170.8
Post-HBsAg WP NAT yield	0.0	0.0	0.0	0.0	0.0	0.0
Post NAT WP residual risk	0.0	0.0	0.0	0.0	0.0	0.0
HBsAg+/DNA-	2.4	0.0	0.3	4.8	0.0	0.6
OBI NAT yield	7.8	7.4	7.0	4.5	4.2	4.0
OBI NAT residual risk	2.5	2.3	2.2	5.8	5.5	5.2
total infectivity risk	1505.9	20.0	186.3	1505.9	20.0	186.3

LPD + RPT = lapsed plus repeat

Supplementary Table S6. Percent TT-HBV transmission risk per million donations in eight HBV infection stages estimated with Weusten WP and OBI risk models as compared to overall risk without screening

South Africa

Infection risk category	First time	Lpd + Rpt	All	First time	Lpd + Rpt	All
	**cobas MPX ID-NAT**			**Ultrio Plus ID-NAT**		
Pre-NAT WP residual risk	0.77%	9.37%	2.15%	1.05%	12.84%	2.95%
Pre-HBsAg WP NAT yield	2.15%	26.21%	6.02%	1.86%	22.73%	5.22%
HBsAg+/DNA+	94.63%	52.57%	87.82%	94.57%	52.57%	87.77%
Post-HBsAg WP NAT yield	0.42%	1.55%	0.62%	0.37%	1.38%	0.55%
Post NAT WP residual risk	0.02%	0.07%	0.03%	0.06%	0.24%	0.10%
HBsAg+/DNA-	0.03%	0.00%	0.03%	0.09%	0.01%	0.07%
OBI NAT yield	1.91%	9.79%	3.20%	1.70%	8.72%	2.84%
OBI NAT residual risk	0.08%	0.44%	0.14%	0.29%	1.51%	0.49%
total infectivity risk	100%	100%	100%	100%	100%	100%
	**cobas MPX MP6-NAT**			**Ultrio Plus MP16-NAT**		
Pre-NAT WP residual risk	1.32%	16.12%	3.70%	1.91%	23.26%	5.34%
Pre-HBsAg WP NAT yield	1.60%	19.46%	4.47%	1.01%	12.32%	2.83%
HBsAg+/DNA+	94.52%	52.56%	87.73%	94.38%	52.55%	87.61%
Post-HBsAg WP NAT yield	0.33%	1.23%	0.49%	0.19%	0.70%	0.28%
Post NAT WP residual risk	0.10%	0.39%	0.16%	0.25%	0.91%	0.37%
HBsAg+/DNA-	0.14%	0.01%	0.12%	0.28%	0.03%	0.23%
OBI NAT yield	1.51%	7.76%	2.53%	0.86%	4.45%	1.45%
OBI NAT residual risk	0.48%	2.47%	0.81%	1.12%	5.78%	1.89%
total infectivity risk	100%	100%	100%	100%	100%	100%

South-East Asia

Infection risk category	First time	Lpd + Rpt	All	First time	Lpd + Rpt	All
	**cobas MPX ID-NAT**			**Ultrio Plus ID-NAT**		
Pre-NAT WP residual risk	0.25%	5.16%	0.76%	0.35%	7.08%	1.04%
Pre-HBsAg WP NAT yield	0.71%	14.45%	2.13%	0.62%	12.54%	1.85%
HBsAg+/DNA+	96.88%	44.39%	92.01%	96.66%	44.22%	91.80%
Post-HBsAg WP NAT yield	0.13%	1.80%	0.27%	0.11%	1.60%	0.24%
Post NAT WP residual risk	0.01%	0.08%	0.01%	0.02%	0.28%	0.04%
HBsAg+/DNA-	0.12%	0.09%	0.11%	0.33%	0.26%	0.32%
OBI NAT yield	1.83%	32.58%	4.50%	1.63%	29.00%	4.01%
OBI NAT residual risk	0.08%	1.45%	0.20%	0.28%	5.03%	0.69%
total infectivity risk	100%	100%	100%	100%	100%	100%
	**cobas MPX MP6-NAT**			**Ultrio Plus MP16-NAT**		
Pre-NAT WP residual risk	0.44%	8.89%	1.31%	0.63%	12.82%	1.89%
Pre-HBsAg WP NAT yield	0.53%	10.73%	1.58%	0.33%	6.79%	1.00%
HBsAg+/DNA+	96.46%	44.06%	91.60%	95.94%	43.65%	91.09%
Post-HBsAg WP NAT yield	0.10%	1.42%	0.22%	0.06%	0.82%	0.12%
Post NAT WP residual risk	0.03%	0.45%	0.07%	0.08%	1.06%	0.16%
HBsAg+/DNA-	0.53%	0.42%	0.52%	1.05%	0.83%	1.03%
OBI NAT yield	1.45%	25.81%	3.57%	0.83%	14.79%	2.04%
OBI NAT residual risk	0.46%	8.22%	1.13%	1.08%	19.24%	2.66%
total infectivity risk	100%	100%	100%	100%	100%	100%

Supplemental table S6 continued

Mediterranean

Infection risk category	First time	Lpd + Rpt	All	First time	Lpd + Rpt	All
	**cobas MPX ID-NAT**			**Ultrio Plus ID-NAT**		
Pre-NAT WP residual risk	0.87%	6.79%	1.82%	1.19%	9.31%	2.49%
Pre-HBsAg WP NAT yield	2.43%	18.99%	5.09%	2.11%	16.47%	4.41%
HBsAg+/DNA+	93.74%	20.26%	80.93%	93.65%	20.24%	80.85%
Post-HBsAg WP NAT yield	0.39%	2.51%	0.72%	0.34%	2.24%	0.64%
Post NAT WP residual risk	0.02%	0.11%	0.03%	0.06%	0.39%	0.11%
HBsAg+/DNA-	0.05%	0.01%	0.04%	0.14%	0.04%	0.12%
OBI NAT yield	2.41%	49.13%	10.89%	2.14%	43.74%	9.69%
OBI NAT residual risk	0.11%	2.19%	0.48%	0.37%	7.58%	1.68%
total infectivity risk	100%	100%	100%	100%	100%	100%
	**cobas MPX MP6-NAT**			**Ultrio Plus MP16-NAT**		
Pre-NAT WP residual risk	1.49%	11.68%	3.13%	2.15%	16.85%	4.51%
Pre-HBsAg WP NAT yield	1.80%	14.10%	3.78%	1.14%	8.93%	2.39%
HBsAg+/DNA+	93.56%	20.22%	80.78%	93.34%	20.16%	80.59%
Post-HBsAg WP NAT yield	0.31%	1.99%	0.57%	0.18%	1.14%	0.33%
Post NAT WP residual risk	0.10%	0.63%	0.18%	0.23%	1.48%	0.42%
HBsAg+/DNA-	0.22%	0.06%	0.19%	0.44%	0.12%	0.39%
OBI NAT yield	1.91%	38.93%	8.63%	1.09%	22.31%	4.94%
OBI NAT residual risk	0.61%	12.39%	2.75%	1.42%	29.01%	6.43%
total infectivity risk	100%	100%	100%	100%	100%	100%

Central-North Europe

Infection risk category	First time	Lpd + Rpt	All	First time	Lpd + Rpt	All
	**cobas MPX ID-NAT**			**Ultrio Plus ID-NAT**		
Pre-NAT WP residual risk	0.19%	6.99%	0.79%	0.26%	9.59%	1.08%
Pre-HBsAg WP NAT yield	0.53%	19.57%	2.21%	0.46%	16.98%	1.92%
HBsAg+/DNA+	98.56%	24.94%	91.99%	98.49%	24.91%	91.93%
Post-HBsAg WP NAT yield	0.00%	0.00%	0.00%	0.00%	0.00%	0.00%
Post NAT WP residual risk	0.00%	0.00%	0.00%	0.00%	0.00%	0.00%
HBsAg+/DNA-	0.03%	0.02%	0.03%	0.10%	0.05%	0.10%
OBI NAT yield	0.66%	46.41%	4.76%	0.59%	41.31%	4.24%
OBI NAT residual risk	0.03%	2.07%	0.21%	0.10%	7.16%	0.73%
total infectivity risk	100%	100%	100%	100%	100%	100%
	**cobas MPX MP6-NAT**			**Ultrio Plus MP16-NAT**		
Pre-NAT WP residual risk	0.33%	12.04%	1.36%	0.47%	17.37%	1.96%
Pre-HBsAg WP NAT yield	0.39%	14.53%	1.64%	0.25%	9.20%	1.04%
HBsAg+/DNA+	98.43%	24.87%	91.87%	98.28%	24.79%	91.72%
Post-HBsAg WP NAT yield	0.00%	0.00%	0.00%	0.00%	0.00%	0.00%
Post NAT WP residual risk	0.00%	0.00%	0.00%	0.00%	0.00%	0.00%
HBsAg+/DNA-	0.16%	0.08%	0.15%	0.32%	0.17%	0.30%
OBI NAT yield	0.52%	36.77%	3.77%	0.30%	21.07%	2.16%
OBI NAT residual risk	0.17%	11.70%	1.20%	0.39%	27.40%	2.81%
total infectivity risk	100%	100%	100%	100%	100%	100%

LPD + RPT = lapsed plus repeat
